# SKAP1 Is a Novel Biomarker and Therapeutic Target for Gastric Cancer: Evidence from Expression, Functional, and Bioinformatic Analyses

**DOI:** 10.3390/ijms241411870

**Published:** 2023-07-24

**Authors:** Lingqin Zhu, Qiongfang Yu, Yuanheng Li, Meng Zhang, Zhiwei Peng, Song Wang, Ziyi Quan, Dian Gao

**Affiliations:** 1 Department of Gastroenterology and Hepatology, Second Affiliated Hospital of Nanchang University, Nanchang 330006, China; lqzhu@email.ncu.edu.cn (L.Z.); qiongfangyu@yeah.net (Q.Y.); ws2773719169@163.com (S.W.); 2 Queen Mary School, Nanchang University, Nanchang 330031, China; 4217121235@email.ncu.edu.cn; 3 Department of Pathogen Biology and Immunology, Medical College of Nanchang University, Nanchang 330006, China; zhangmeng@ncu.edu.cn (M.Z.); zhiweipengjc@ncu.edu.cn (Z.P.); 18070132515@163.com (Z.Q.)

**Keywords:** gastric cancer, SKAP1, JAK/PI3K/AKT axis, proliferation, invasion, migration, immunity

## Abstract

Gastric cancer (GC) is the third leading cause of cancer-related death worldwide. Due to the lack of early symptoms, GC is often diagnosed at an advanced stage when treatment options are limited. There is an urgent need to identify biomarkers for early detection, prognosis evaluation, and targeted treatment of GC. Studies have shown that Src kinase-associated phosphoprotein 1 (SKAP1) promotes cell proliferation and invasion and is associated with poor prognosis in colorectal cancer, malignant fibrous histiocytoma, and breast cancer. However, the role and mechanism of SKAP1 in GC are unclear. Here, analyses of multiple databases and experiments revealed that SKAP1 expression was higher in GC than in adjacent normal tissues. The Cancer Genome Atlas data showed that high SKAP1 expression was associated with poor GC prognosis. SKAP1 expression was higher in GC than in normal gastric epithelial cells. SKAP1 silencing reduced the proliferation, migration and invasion of the GC cell lines MKN45 and HGC27. Rescue experiments suggest that SKAP1 may promote GC progression by activating JAK1/PI3K/AKT signaling and regulating GC cell proliferation, invasion, migration, and other functions. Bioinformatics analysis revealed that SKAP1 was associated with immune cell infiltration and checkpoint expression in GC. High SKAP1 expression was associated with poorer immunotherapy outcomes, suggesting its potential as a predictive biomarker of GC immunotherapy efficacy. In summary, SKAP1 is overexpressed in GC, where it promotes cell proliferation, invasion and migration and is associated with poor prognosis and poor immunotherapy outcomes. SKAP1 may represent a biomarker and therapeutic target in GC and regulates cellular functions through JAK1/PI3K/AKT signaling.

## 1. Introduction

Gastric cancer (GC) is the fourth leading cause of death among malignant tumors globally; accounting for 7.7% of all cancer-related deaths, after lung cancer, colorectal cancer, and liver cancer. It poses a severe threat to human physical and mental health and presents significant social and economic burdens [1]. Despite the effective control of *Helicobacter pylori* infection in recent decades and significant advances in early diagnosis and treatment, GC remains associated with poor prognosis and a low 5-year survival rate [2,3]. Consequently, elucidating the molecular mechanisms of GC is urgently needed to develop precise diagnostics and therapies, especially for patients with advanced and inoperable disease.

Abnormal gene expression in GC may be involved in tumor occurrence and progression, leading to poor outcomes. [4] Through bioinformatics analysis and a comprehensive literature review, we identified a gene called SKAP1 (Src kinase-associated phosphoprotein 1), which may be associated with the occurrence and development of GC. SKAP1, also known as SKAP55 or HEL-S-81p, belongs to the Src kinase family and encodes an intracellular T-cell adaptor protein [5]. Although it lacks enzymatic activity, it possesses modular domains that recruit other proteins. SKAP proteins include SKAP1 [6] or SKAP55 [7] and SKAP2 [8] or SKAP-HOM/SKAP-55R [9,10]. They share dimerization (DM), pleckstrin homology (PH) and C-terminal Src homology 3 (SH3) domains, with 44% sequence identity, especially in the PH and SH3 domains [9,10]. In various cancers, such as non-small cell lung cancer and breast cancer, both SKAP1 and SKAP2 are upregulated, promoting tumor progression, although their underlying mechanisms remain unclear [11,12,13,14]. Moreover, SKAP1 has been implicated in the regulation of cell cycle progression and proliferation. It has been identified as a substrate of polo-like kinase 1 (PLK1). PLK1 controls crucial cellular processes, including mitosis, spindle assembly, chromosome separation, DNA replication, cytokinesis, and meiosis. Overexpression of PLK1 has been observed in neuroblastoma, colorectal cancer, and GC and correlates with poor prognosis. PLK1 phosphorylates SKAP1 at serine 31, and together, they interact to regulate T-cell proliferation [15,16,17,18,19,20]. Furthermore, increased expression of SKAP1 has been detected in colorectal cancer tissues and cells, where it not only plays a pivotal role in promoting cell invasion and metastasis but also serves as a prognostic marker for poor outcomes in colorectal cancer patients [21].

Some studies have shown that SKAP1 is involved in immune responses. Decreased expression of SKAP1 impairs T-cell tumor responses, resulting in decreased tumor immunosurveillance and an increased risk of endometrial cancer [22]. Deleting the SH3 or DM domain of SKAP1 impairs SLP-76 cluster formation and reduces T-cell binding to intercellular adhesion molecule-1 and antigen-presenting cells [23,24,25]. SKAP1 and ankle protein bind lymphocyte function-associated antigen 1 alpha and beta chains, activating T-cell transendothelial migration [26]. SKAP1 also activates lymphocyte function-associated antigen 1 through T-cell receptor-mediated “inside-out” signaling by forming a SKAP1-RapL-Rap1 complex, stimulating lymphocyte adhesion and homing [24,27].

Despite these findings, the specific role and regulatory mechanism of SKAP1 in GC development and progression remain unclear. In this study, we assessed the expression levels of SKAP1 in GC tissues and adjacent normal tissues, analyzed the relationship between SKAP1 expression levels and the clinicopathological parameters of GC patients, and evaluated its prognostic value. We also investigated the expression of SKAP1 in GC cells and its role in cell proliferation, migration, and invasion, as well as its potential mechanisms. Furthermore, we examined the involvement of SKAP1 in GC immunity. The findings from this study may help to identify a new tumor biomarker and provide new insights into diagnosis and treatment.

## 2. Results

### 2.1. High Expression of SKAP1 in GC Tissue

**Figure 1:**
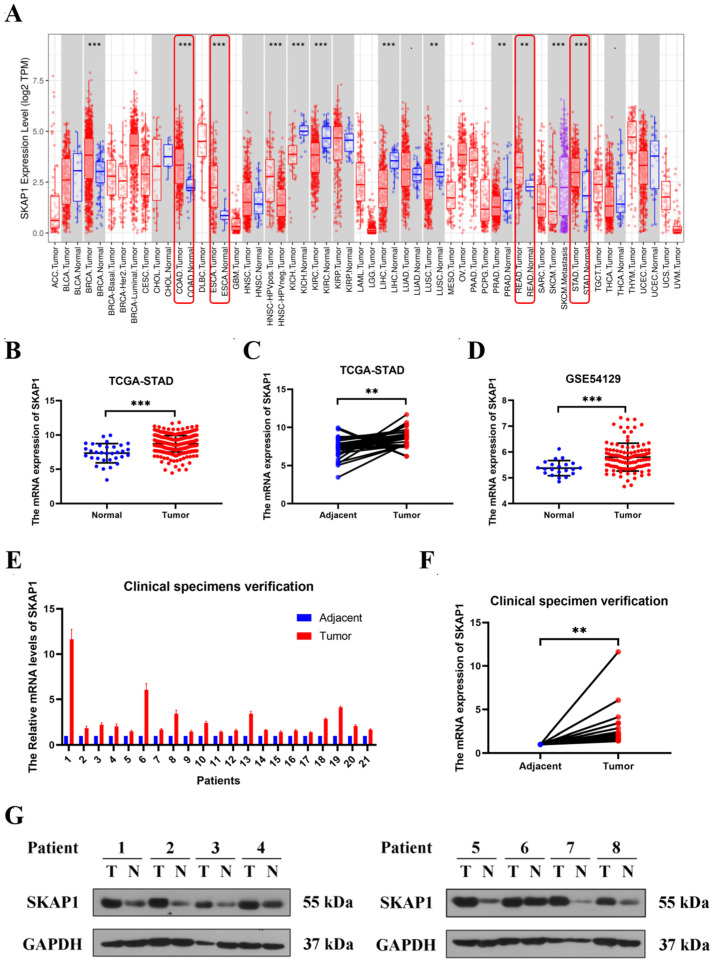
SKAP1 is overexpressed in GC tissue. (**A**) SKAP1 expression across different cancers from the TIMER database, with tumor tissue in red and normal tissue in blue. Gastrointestinal tumors are marked by red boxes. (**B**,**C**) SKAP1 expression in GC based on the TCGA dataset. (**B**) shows SKAP1 expression in GC and normal tissue and (**C**) shows SKAP1 expression in paired GC and adjacent tissue from TCGA. (**D**) SKAP1 expression in GC based on the GEO dataset (GSE54129). (**E**,**F**) SKAP1 mRNA levels in 21 pairs of GC and adjacent tissues. (**G**) SKAP1 protein levels in GC and adjacent tissues by Western blot analysis (8 representative samples). ** *p* < 0.01, *** *p* < 0.001.

To elucidate the expression levels of SKAP1 in various cancer types, we conducted an analysis using Tumor Immune Estimation Resource (TIMER). Our results demonstrated a marked upregulation of SKAP1 in digestive tract tumors, including stomach adenocarcinoma (STAD), esophageal carcinoma (ESCA), colon adenocarcinoma (COAD), and rectal adenocarcinoma (READ), compared to the corresponding controls (*p* < 0.05, Figure 1A). These findings strongly indicate the potential involvement of SKAP1 in the development and progression of digestive tract tumors. To further explore the role of SKAP1 in GC, we analyzed the The Cancer Genome Atlas (TCGA) GC dataset and Gene Expression Omnibus (GEO) dataset (GSE54129) and found that SKAP1 was significantly upregulated in GC tissues vs. normal tissues (*p* < 0.05, Figure 1B–D). This finding reveals that SKAP1 is possibly associated with tumor progression.

To validate these findings, we collected 21 clinicopathologically confirmed GC tissue samples and performed Western blotting and qPCR experiments to detect SKAP1 expression levels. The results showed that the expression of SKAP1 in GC tissues was significantly higher than that in adjacent tissues (*p* < 0.01, Figure 1E–G). These findings support the hypothesis that SKAP1 is involved in GC development and progression.

### 2.2. Relationship between SKAP1 and Clinicopathological Parameters and Prognosis of GC Patients

**Figure 2:**
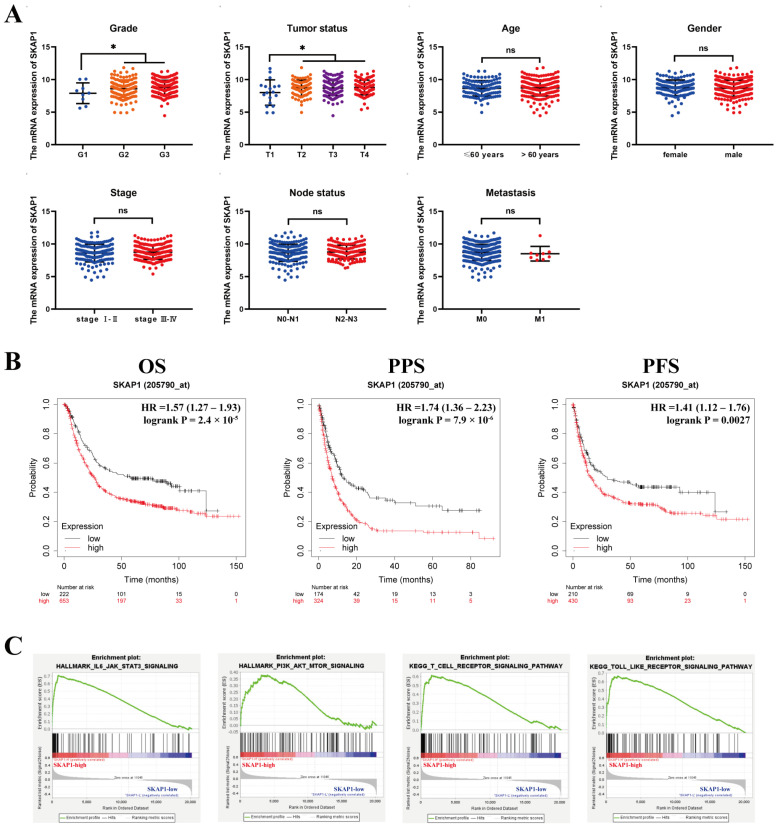
Bioinformatic analysis of SKAP1 expression and its association with GC prognosis and potential mechanisms. (**A**) SKAP1 expression and its correlation with tumor grade, tumor status, age, gender, stage and metastasis (N, M stage) based on the TCGA-STAD dataset. (**B**) SKAP1 expression and its correlation with overall survival, postprogression survival, and progression-free survival in GC patients from Kaplan–Meier Plotter. The red survival curve belongs to the SKAP1 high-expression group, while the black one belongs to the SKAP1 low-expression group. (**C**) GSEA enrichment pathways (JAK/STAT signaling, PI3K/AKT signaling, T-cell receptor signaling, Toll-like receptor signaling) in datasets with high or low SKAP1 expression. The statistical significance is indicated as ns (no significance), * *p* < 0.05.

In the present study, we investigated the relationship between SKAP1 expression and clinicopathological parameters as well as the prognosis of GC patients. Using the TCGA database, we found that SKAP1 expression was significantly associated with the tumor stage (T stage) and tumor differentiation grade (grade) of patients but not with patient age, gender, stage, or metastasis (N, M) (Figure 2A). Moreover, patients with high SKAP1 expression had significantly shorter overall survival (OS) (HR = 1.57, 95% CI = 1.27~1.93, *p* = 2.4 × 10^−5^), postprogression survival (PPS) (HR = 1.74, 95% CI = 1.36~2.23, *p* = 7.9 × 10^−6^), and progression-free survival (PFS) (HR = 1.41, 95% CI = 1.12~1.76, *p* = 0.0027) than those with low SKAP1 expression (*p* < 0.05, Figure 2B). The above results suggest that high SKAP1 expression may be an adverse prognostic factor in GC.

### 2.3. Bioinformatics Prediction of the Possible Mechanism by Which SKAP1 Regulates GC Progression

To elucidate the mechanism by which SKAP1 regulates GC progression, we conducted Gene Set Enrichment Analysis (GSEA) on a cohort of patients stratified based on SKAP1 expression levels (high vs. low with the median SKAP1 expression value as the cutoff). Our analysis identified significant enrichment of the JAK-STAT pathway and PI3K-AKT pathway in the high-SKAP1 expression group, as demonstrated graphically in Figure 2C. Moreover, high SKAP1 expression was correlated with a range of proliferation-associated biological processes, including MYC targets, E2F targets, G2M cell cycle checkpoints, spindle formation, and DNA repair, among others (Figure S1A–F). In addition, our findings indicate the potential involvement of SKAP1 in various immune-related signaling pathways, such as T-cell receptor signaling, Toll-like receptor signaling (Figure 2C), the intestinal immune network for IgA production, antigen processing and presentation, natural killer cell-mediated cytotoxicity, primary immunodeficiency, and B-cell receptor signaling (Figure S1G–K).

### 2.4. Effect of Silencing SKAP1 on GC Cell Biology

**Figure 3:**
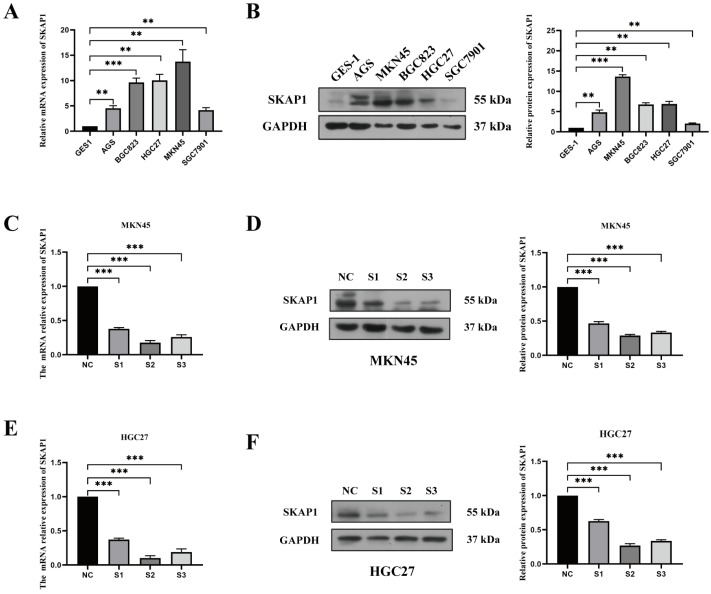
qPCR and Western blot analysis of SKAP1 expression and siRNA knockdown efficiency in GC cell lines. (**A**) SKAP1 mRNA levels in normal gastric epithelial cells (GES-1) and GC cells. (**B**) SKAP1 protein levels in normal gastric epithelial cells and GC cells. (**C**,**F**) qPCR and WB validation of SKAP1 knockdown efficiency in MKN45 cells (**C**,**D**) and HGC27 cells (**E**,**F**). Negative control (NC) cells represent cells transfected with a scrambled siRNA, while S1, S2, and S3 represent siSKAP1-1-, siSKAP1-2-, and siSKAP1-3-transfected cells, respectively. ** *p* < 0.01, *** *p* < 0.001. The experiment was repeated independently three times.

To explore the impact of SKAP1 on the biological behavior of GC cells, we first examined the RNA and protein levels of SKAP1 in gastric mucosal epithelial cells (GES-1) and GC cells via Western blotting and qPCR experiments. Our findings revealed substantially higher expression of SKAP1 in GC cells than in normal gastric mucosal epithelial cells (Figure 3A,B). As such, we selected MKN45 and HGC27 cell lines with high SKAP1 expression for transfection of siRNA targeting SKAP1. Quantification of SKAP1 transcript and protein levels following transfection with three different siRNAs, each of which specifically targeted binding sites on SKAP1, revealed a significant decrease in SKAP1 expression compared to that in the negative control (NC) group (Figure 3C–F). Among the siRNAs tested, siSKAP1-2 and siSKAP1-3 had greater silencing efficacy than siSKAP1-1, and these two siRNAs were used for subsequent experiments.

**Figure 4:**
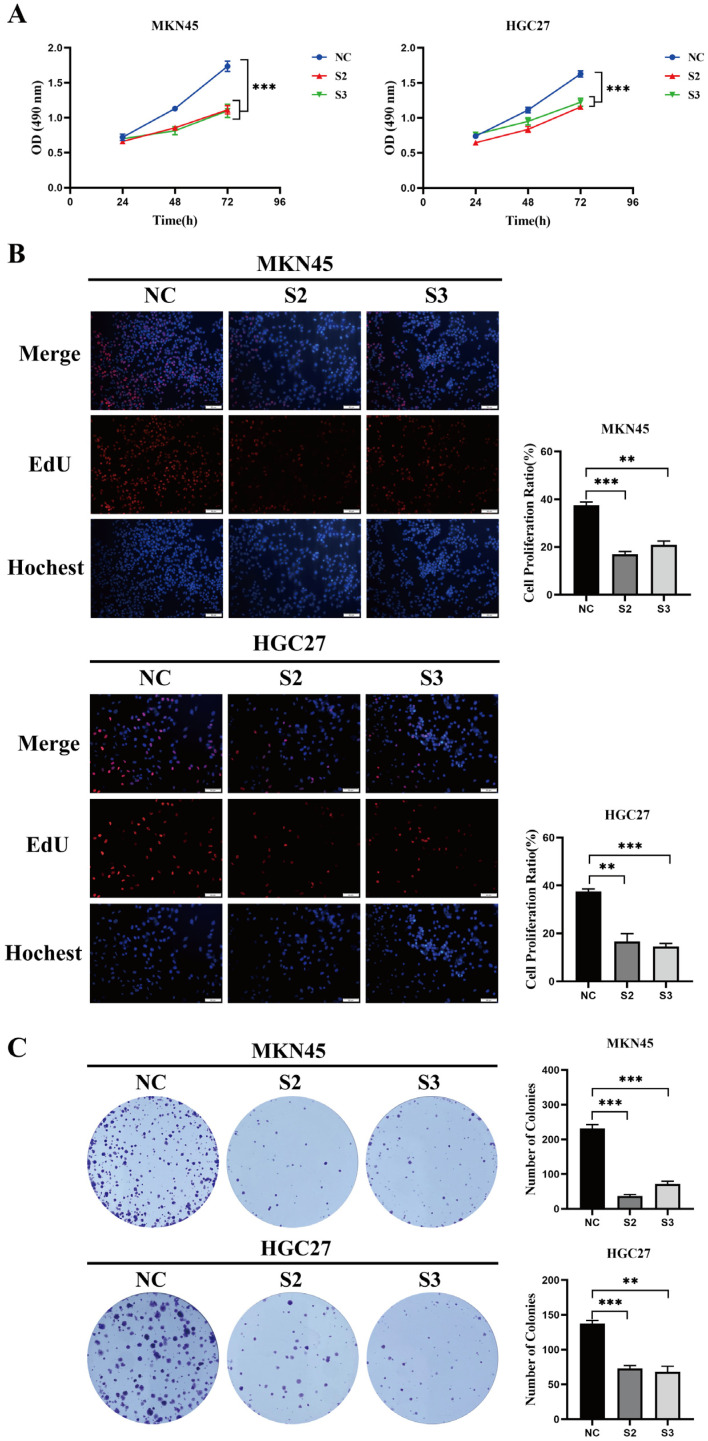
Inhibition of SKAP1 suppresses GC cell proliferation. (**A**) MTS assay showing the growth curve of MKN45 and HGC27 cells; (**B**) EdU assay detecting the proliferation rate of MKN45 and HGC27 cells (200×); (**C**) colony formation assay evaluating the colony-forming ability of MKN45 and HGC27 cells. NC represents cells transfected with a scrambled siRNA, while S1, S2, and S3 represent siSKAP1-1-, siSKAP1-2-, and siSKAP1-3-transfected cells, respectively. ** *p* < 0.01, *** *p* < 0.001. The experiments were repeated independently three times with similar results.

**Figure 5:**
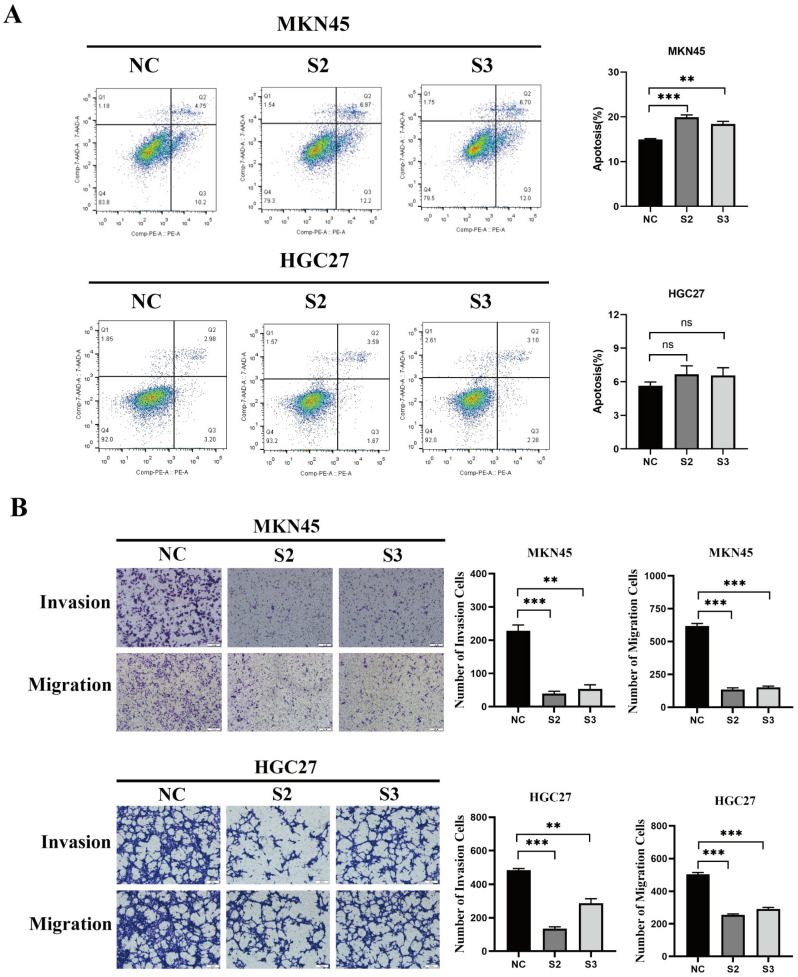
Silencing SKAP1 induces apoptosis and inhibits the invasion/migration of GC cells. (**A**) Flow cytometry analysis detecting the apoptotic rate of MKN45 and HGC27 cells; (**B**) Transwell migration and invasion assays assessing the invasion and migration abilities of MKN45 and HGC27 cells. NC represents cells transfected with a scrambled siRNA, while S1, S2, and S3 represent siSKAP1-1-, siSKAP1-2-, and siSKAP1-3-transfected cells, respectively (100×). The statistical significance is indicated as ns (no significance), ** *p* < 0.01, *** *p* < 0.001. All experiments were repeated three times with similar results.

MTS cell proliferation experiments revealed that the proliferation ability of MKN45 and HGC27 cells was significantly lower in the SKAP1 knockdown group than in their respective NC groups (*p* < 0.05, Figure 4A). EdU is a thymidine analog that can replace thymidine (T) during DNA replication and incorporate into the growing DNA molecule. Based on the specific reaction between Apollo^®^ fluorescent dye and EdU, DNA replication activity can be directly and accurately detected. Similarly, EdU assays demonstrated that DNA synthesis activity was considerably reduced in cells in the SKAP1 silencing group compared to the NC group (Figure 4B), while colony formation assays demonstrated that the number of colonies generated by cells with SKAP1 knockdown expression was significantly less than the number generated by NC group cells (Figure 4C). In addition, we detected cell apoptosis using an Annexin V-PE/7AAD apoptosis detection kit. The results indicated a significant increase in cell apoptosis after SKAP1 knockdown in MKN45 cells, whereas SKAP1 silencing did not have a significant impact on apoptosis levels in HGC27 cells (Figure 5A). These findings suggest that the effect of SKAP1 on cell apoptosis is not consistent in different GC cell lines, possibly due to heterogeneity arising from different cell sources. Additionally, Transwell assays demonstrated significant suppression of the invasive and migratory capabilities of both MKN45 and HGC27 cells following SKAP1 knockdown (*p* < 0.05, Figure 5B). Overall, our results indicate that silencing SKAP1 effectively inhibits the proliferation, invasion, and migration of GC cells, underscoring the potential value of SKAP1 as a diagnostic and therapeutic target for GC.

### 2.5. SKAP1 Regulates the JAK1/PI3K/AKT Signaling Axis in GC Cells

**Figure 6:**
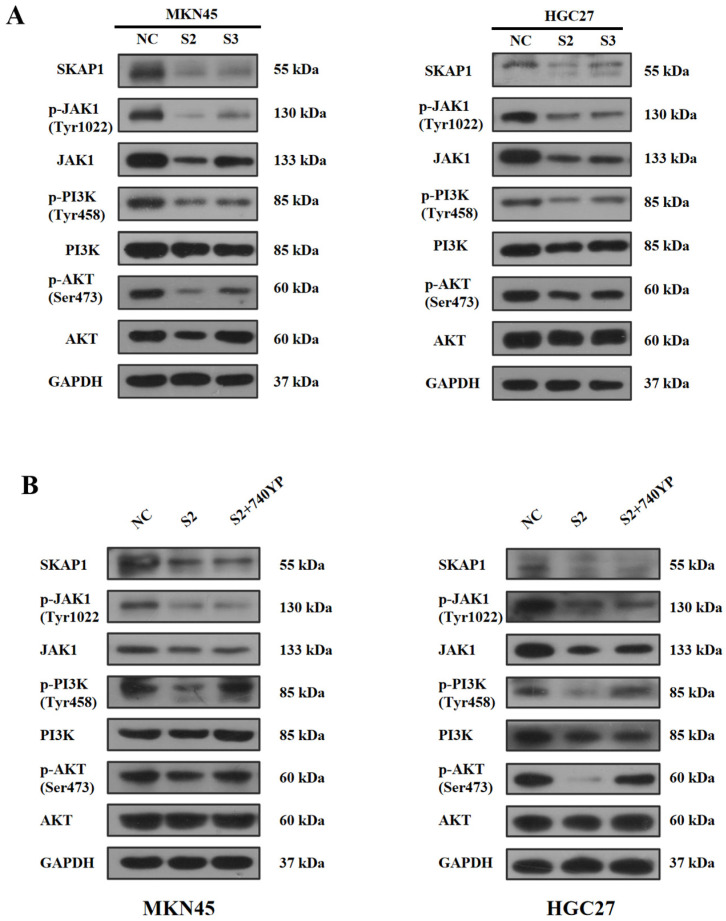
SKAP1 modulates the JAK1/PI3K/AKT signaling pathway in GC cells. (**A**) Western blot analysis measuring the levels of JAK1, PI3K, AKT, and their phosphorylated proteins after SKAP1 knockdown. (**B**) Western blot analysis detecting the levels of JAK1, PI3K, AKT, and their phosphorylated proteins after treatment with the PI3K activator 740Y-P (20 µg/mL). NC represents cells transfected with a scrambled siRNA, while S2 and S3 represent siSKAP1-2- and siSKAP2-3-transfected cells, respectively. S2+740Y-P represents siSKAP1-2+PI3K activator-treated cells. All experiments were repeated three times.

The previous GSEA enrichment analysis revealed significant enrichment of the JAK/STAT and PI3K/AKT pathways in the high SKAP1 expression group. To further investigate the regulatory mechanism underlying the effect of SKAP1 on GC cells, we conducted quantitative measurement of the mRNA and protein levels of key molecules in these pathways. Subsequent qPCR analysis demonstrated downregulation of JAK1 mRNA levels in MKN45 cells, as well as decreased mRNA levels of PIK3CA and PIK3CD, following SKAP1 silencing (Figure S2). As JAK can activate the PI3K-AKT signaling pathway directly without STAT [28], we inferred that SKAP1 can modulate the JAK/PI3K/AKT signaling pathway to influence the biological function of GC cells. Further Western blot analysis confirmed that silencing SKAP1 led to significantly reduced protein expression of JAK1 and P-JAK1, as well as decreased expression of P-PI3K and P-AKT compared to that in the control group (Figure 6A).

To explore whether SKAP1 regulates the biological behavior of GC cells through the JAK1/PI3K/AKT signaling axis, we added a PI3K agonist (740Y-P, 20 µg/mL) to SKAP1-siRNA-transfected GC cells and measured the protein expression levels by Western blotting. The results indicated that silencing SKAP1 led to a significant reduction in JAK1 and P-JAK1 expression, as well as decreased expression of P-PI3K and P-AKT relative to total PI3K and AKT. However, after adding the PI3K agonist, the protein expression levels of P-PI3K and P-AKT were increased, while JAK1 and P-JAK1 levels remained unchanged (Figure 6B). These results suggest that SKAP1 may modulate the JAK1/PI3K/AKT signaling axis in GC cells and targeting that this pathway could be a potential therapeutic strategy for GC treatment.

### 2.6. Effect of a PI3K Agonist on the SKAP1-Regulated Biological Behavior of GC Cells

To further explore whether SKAP1 regulates GC cell biological behavior via the JAK1/PI3K/AKT signaling axis, we added a PI3K agonist (740Y-P, 20 µg/mL) to SKAP1-siRNA-transfected GC cells and analyzed changes in their biological behavior. Specifically, we examined MKN45 and HGC27 GC cells in the NC group, S2 (siSKAP1-2) group, and S2+740Y-P group.

**Figure 7:**
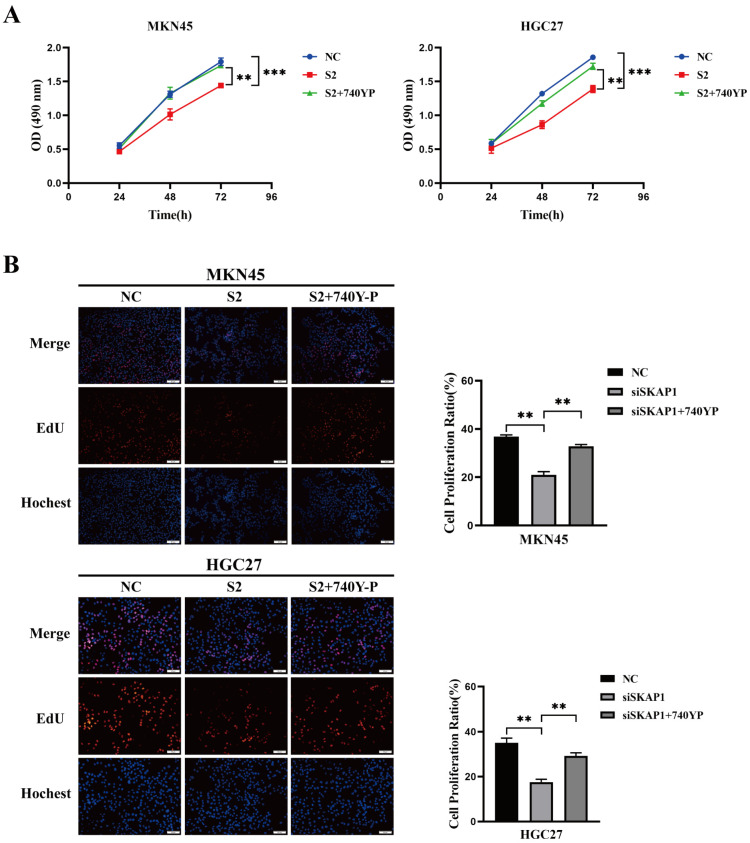

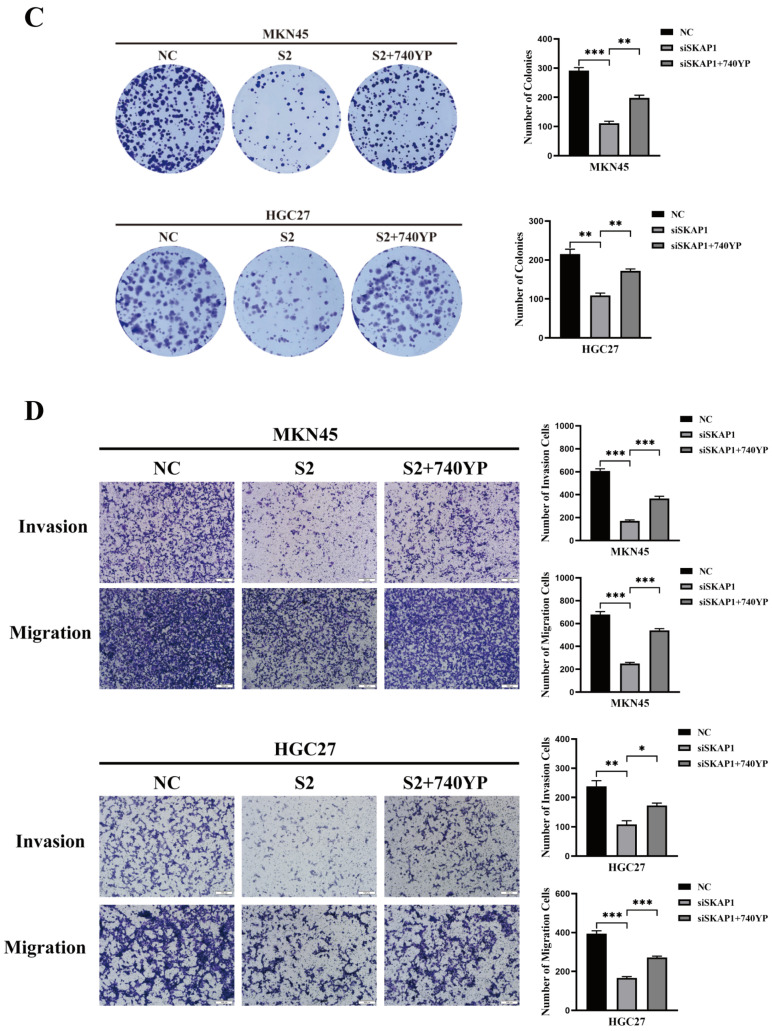
PI3K activator alleviates the inhibitory effect of silencing SKAP1 on the biological behavior of GC cells. (**A**) MTS assay detecting the proliferation ability of MKN45 and HGC27 cells in each group; (**B**) EdU assay measuring the proliferation rate of MKN45 and HGC27 cells in each group (200×); (**C**) colony formation assay evaluating the colony-forming ability of MKN45 and HGC27 cells in each group; (**D**) Transwell migration and invasion assays assessing the migration and invasion abilities of MKN45 and HGC27 cells in different groups (100×). NC indicates cells transfected with a scrambled siRNA, while S2 and S2+740Y-P represent siSKAP1-2 transfected cells and siSKAP1-2+PI3K activator-treated cells, respectively. * *p* < 0.05, ** *p* < 0.01, *** *p* < 0.001. All experiments were repeated three times with similar results.

MTS cell proliferation assay results showed that the proliferation ability of MKN45 and HGC27 cells was significantly lower in the SKAP1-silenced group than in the control group. However, this reduced proliferation ability was partially recovered after adding 740Y-P (20 µg/mL) (Figure 7A). Similarly, the EdU assay showed that the DNA synthesis activity of the SKAP1 knockdown group was significantly lower than that of the control group but increased significantly in the siSKAP1-2+740Y-P group compared to the siSKAP1-2 group (Figure 7B). Additionally, the colony formation assay showed that the number of colonies formed by cells with low SKAP1 expression was significantly fewer than that of the control group but increased significantly in the siSKAP1-2+740Y-P group compared to the siSKAP1-2 group (Figure 7C). Furthermore, Transwell invasion and migration assays revealed that the invasion and migration ability of MKN45 and HGC27 cells decreased significantly after SKAP1 knockdown. However, the cells in the siSKAP1-2+740Y-P group exhibited significantly enhanced invasion and migration ability compared to the siSKAP1-2 group cells (Figure 7D). These results suggest that SKAP1 may modulate GC cell biological behavior through the JAK1/PI3K/AKT signaling axis.

### 2.7. Bioinformatics Analysis of the Relationship between SKAP1 Expression and Immune Infiltration in GC

**Figure 8:**
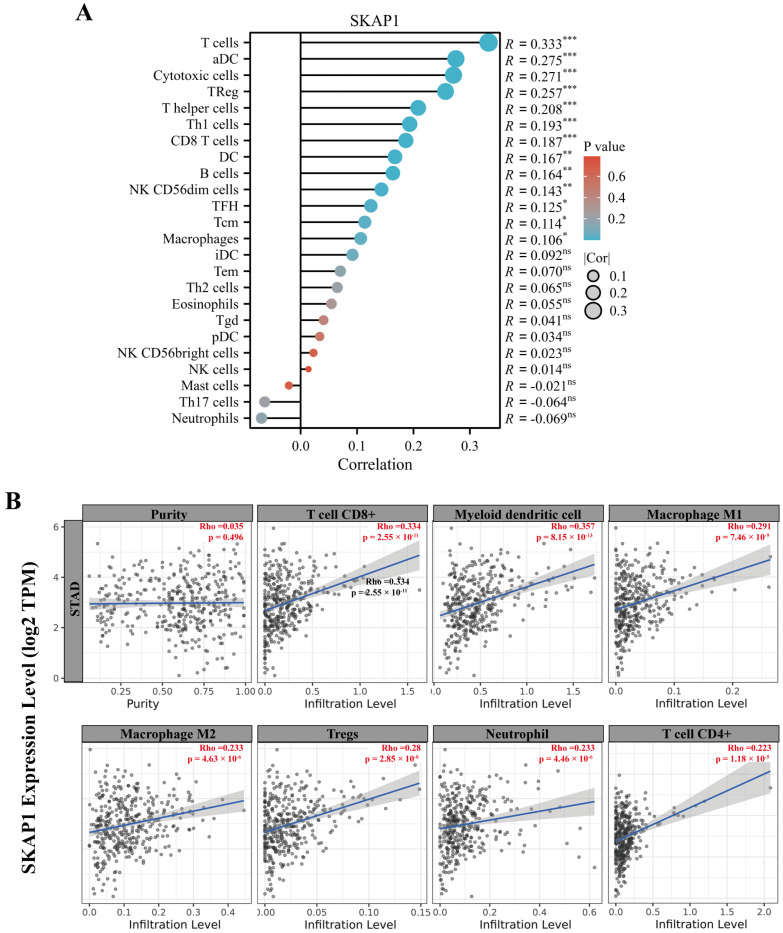
SKAP1 expression correlates with immune cell infiltration in GC. (**A**) Correlation between SKAP1 expression and immune cell infiltration in the GC TME based on the ssGSEA algorithm. (**B**) The purity-corrected partial Spearman’s rho value was used to evaluate the correlation between SKAP1 expression and immune cell infiltration in the GC tumor microenvironment after adjusting for tumor purity using the TIMER database. Each point represents a patient with GC, and the line represents the trendline. * *p* < 0.05, ** *p* < 0.01, *** *p* < 0.001, ns represents no significance.

**Figure 9:**
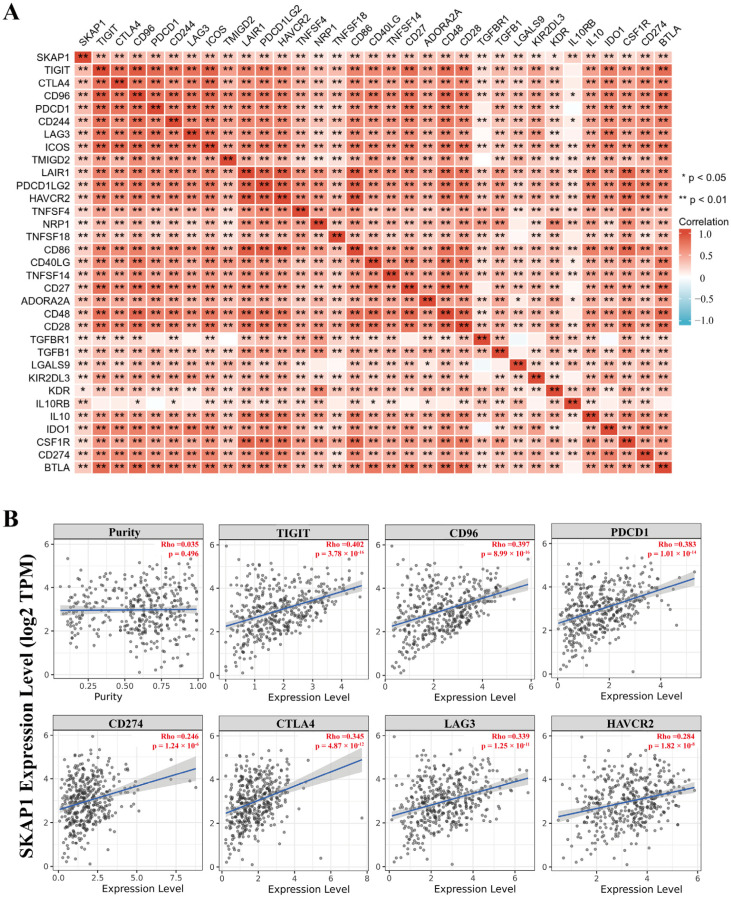
SKAP1 is significantly positively correlated with multiple immune checkpoints in GC. (**A**) Analysis of the correlation between SKAP1 expression and multiple immune checkpoints based on the TCGA-STAD dataset; (**B**) The purity-corrected partial Spearman’s rho value was used to evaluate the correlation between SKAP1 expression and several common immune checkpoints (TIGIT, CD96, PD1, PD-L1, CTLA4, LAG3, TIM3) in GC after adjusting for tumor purity using the TIMER database. Each point represents a patient with GC, and the line represents the trendline.

**Figure 10:**
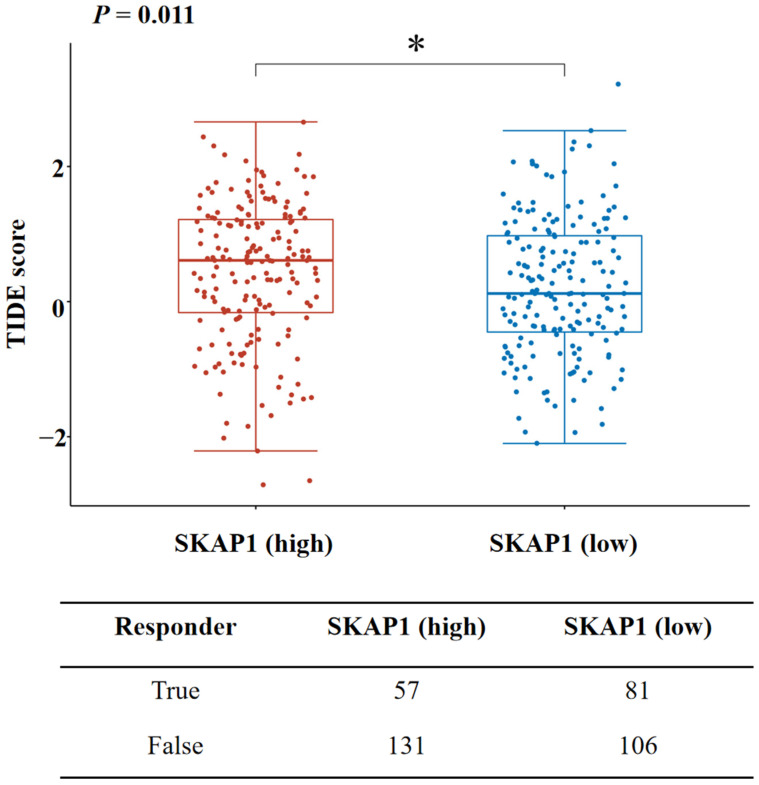
SKAP1 expression correlates with TIDE score in GC tissues. * *p* < 0.05.

To investigate the relationship between SKAP1 expression and immune infiltration in GC, we used R language and the TIMER database to analyze the TCGA-STAD dataset. SKAP1 expression levels were positively correlated with the infiltration levels of various immune cells, including T cells, cytotoxic cells, DCs, and Treg cells (Figure 8A,B). Moreover, SKAP1 expression showed significant positive correlations with multiple immune cell markers (Table S3). We also used R language to examine the association between SKAP1 expression and several immune checkpoint molecules and found that SKAP1 expression was significantly positively correlated with all of them (Figure 9A). This result was further confirmed by the TIMER 2.0 database, which showed that SKAP1 expression had significant positive correlations with common immune checkpoint molecules, such as TIGIT (R = 0.402), CD96 (R = 0.397), PD1 (R = 0.383), PD-L1 (R = 0.246), CTLA4 (R = 0.345), LAG3 (R = 0.339), and TIM-3 (R = 0.284) (Figure 9B). Furthermore, we stratified the patients in the TCGA-STAD dataset into high and low SKAP1 expression groups based on the median expression value and calculated their TIDE scores. We found that the TIDE score was significantly higher in the high SKAP1 expression group than in the low SKAP1 expression group (*p* = 0.011) (Figure 10). These results indicate that high SKAP1 expression is associated with an impaired immunotherapy response in GC patients and suggest its potential as a predictive biomarker for GC immunotherapy efficacy.

## 3. Discussion

Biomarker discovery has been widely used as a strategy to improve the diagnosis, prognosis evaluation, recurrence prediction, and treatment response of various diseases, including cancer. By acting as indicators of normal biological processes, pathological processes, or pharmacological responses to therapeutic interventions, biomarkers play a crucial role in improving patient outcomes [29]. In this study, we aimed to investigate the potential role of SKAP1 in the development of GC and its underlying molecular mechanisms. Our findings demonstrated that SKAP1 showed high expression in GC tissues and was associated with poor prognosis. We propose that SKAP1 may facilitate GC development by promoting GC cell proliferation, invasion, and migration through activation of the JAK1/PI3K/AKT pathway, making it a possible biomarker for GC.

SKAP1 was initially discovered as a novel adaptor protein that interacts with the tyrosine kinase Fyn [7]. Subsequent studies revealed that SKAP1 promotes cancer progression in breast cancer [14], malignant fibrous histiocytoma [13], and colorectal cancer [21], but its role in GC remains unclear. Through joint analysis of multiple databases, we found that SKAP1 is highly expressed in multiple tumors, particularly digestive tract tumors. We further analyzed SKAP1 expression in GC and found that its expression level was significantly higher than that in normal gastric epithelial tissue. The results of Western blotting and qRT–PCR tests on 21 GC samples were consistent with the bioinformatics analysis results. Additionally, high SKAP1 expression was found to be associated with the T stage and grade classification of GC patients. Hence, we believe that SKAP1 plays a similar role to that reported in previous studies: it promotes GC progression and serves as an adverse prognostic factor for patients.

GSEA analysis suggested that high expression of SKAP1 was significantly correlated with proliferation-related biological processes and inflammatory responses. SKAP1-deficient mice displayed reduced proinflammatory cytokine IL-17 levels, leading to decreased inflammation and heightened resistance to collagen-induced arthritis [30]. Literature reports also state that SKAP1 promotes the growth and invasion of colorectal cancer cells, affecting the occurrence and development of colorectal cancer [21]. In this study, multiple proliferation experiments showed that silencing SKAP1 significantly inhibited the proliferation of MKN45 and HGC27 GC cells and markedly suppressed their invasion and migration. These results align with the findings from previous GSEA analysis and reports, indicating that SKAP1 may play a vital role in promoting the proliferation, invasion, and migration of GC cells in GC development.

Furthermore, GSEA analysis revealed significant enrichment of the JAK-STAT pathway and PI3K-AKT pathway in the high SKAP1 expression group. The PI3K/AKT signaling pathway is one of the most critical intracellular signaling pathways controlling basic cellular functions, including but not limited to cellular proliferation, invasion, metabolism, movement, stress response, and treatment response [31,32]. The JAK-STAT signaling pathway is also crucial in cancer pathogenesis, regulating cell differentiation, proliferation, apoptosis, and the immune response [33]. In this study, we found that high SKAP1 expression was significantly correlated with the activation of the JAK1/PI3K/AKT pathway. Subsequent cell experiments confirmed that silencing SKAP1 in GC cells decreased the phosphorylation levels of JAK1, PI3K, and AKT, thereby significantly suppressing the proliferation, migration, and invasion of GC cells. These findings suggest that SKAP1 may promote GC development by activating the JAK1/PI3K/AKT pathway.

Moreover, SKAP1 has been implicated in several immune-related signaling pathways, such as T-cell receptor signaling and Toll-like receptor signaling. During tumor development, numerous genomic changes often occur, leading to the recognition of tumor cell surface antigens by the immune system and inducing an immune response [34,35]. Immune cells present in the tumor microenvironment, such as NK cells, DCs, macrophages, central granulocytes, T cells, B cells, and other cells involved in innate and adaptive immunity, play critical roles in cancers [36,37,38,39,40,41]. We found a significant correlation between SKAP1 expression and the infiltration of various immune cells, suggesting its potential role in immune infiltration within the tumor microenvironment and its influence on tumor development and clinical prognosis. Additionally, GC cells can alter immune checkpoint molecule expression to avoid immune cell-mediated surveillance and death. SKAP1 expression is positively correlated with the expression levels of immune checkpoint molecules such as PD-1, TIM-3, and LAG3 in GC tissues, and we speculate that inhibiting SKAP1 expression may restore the antitumor immune response and promote immune-mediated malignant cell clearance by inhibiting multiple immune checkpoints. The TIDE score is a comprehensive evaluation system that assesses both T lymphocyte dysfunction and immune inhibitory factor effects on tumor rejection and is considered to be an effective predictor of the efficacy of immune checkpoint inhibitors. In this study, the TIDE score was found to be significantly higher in the SKAP1 high-expression group than in the SKAP1 low-expression group, indicating a correlation between high SKAP1 expression and poor immunotherapy outcomes in GC patients, suggesting its potential as a predictive biomarker for GC immunotherapy efficacy.

Our study’s findings suggest that SKAP1 may serve as a potential biomarker and therapeutic target for GC. Nevertheless, further studies are required to determine the underlying molecular mechanisms of SKAP1 in GC development and to develop more effective targeted therapies for GC patients. Future research may explore SKAP1-targeted therapeutic strategies in preclinical models and evaluate their efficacy and safety in clinical trials.

## 4. Materials and Methods

### 4.1. Expression, Prognosis and Clinicopathological Data Analysis

We downloaded SKAP1 expression data for various tumors from TIMER 2.0 (http://timer.cistrome.org/, accessed on 4 July 2021). We obtained TCGA-STAD, which contains expression values and clinicopathological parameters, from the UCSC Xena Data Browser (https://xenabrowser.net/datapages/, accessed on 21 July 2021), and the GC dataset (GSE54129) from the GEO database (https://www.ncbi.nlm.nih.gov/geo/, accessed on 23 July 2021). These datasets were used to analyze the differences in SKAP1 expression between gastric cancer and adjacent tissues and its correlation with clinicopathological parameters. Moreover, the Kaplan–Meier Plotter website was used to evaluate the prognosis of GC patients by analyzing OS, PPS, and PFS.

### 4.2. Tissues and Cells

All experimental protocols and methods were approved by the Ethics Committee of the Second Affiliated Hospital of Nanchang University and conducted in strict accordance with the Declaration of Helsinki. Written informed consent was obtained from all participants prior to enrollment in the study. GC and adjacent tissue samples were collected from 21 patients who underwent GC surgery. None had received preoperative radiotherapy, chemotherapy, or immunotherapy. The normal human gastric epithelial cell line GES-1 and GC cell lines AGS, BGC823, HGC27, MKN45, and SGC7901 were maintained in our laboratory.

### 4.3. Real-Time Quantitative Reverse Transcription-PCR

RNA was extracted from GC and adjacent tissues using TRIzol reagent (Invitrogen, Carlsbad, CA, USA) and reverse transcribed into cDNA using an RNA reverse transcription kit (Thermo Scientific, Waltham, MA, USA). We performed quantitative PCR using a qPCR kit (Takara SYBR Premix Ex Taq I, Tokyo, Japan) with primers (see Table S1) synthesized by Sangon Biotech (Shanghai, China). We used the 2^−ΔΔCT^ method to analyze the data and normalized SKAP1 expression levels with the endogenous GAPDH mRNA level.

### 4.4. Protein Extraction, Quantification, and Immunoblot Analysis

Proteins were extracted from tissues or cells using RIPA buffer (Sigma-Aldrich, Livonia, MI, USA) containing protease and phosphatase inhibitors (Focus, Shanghai, China). Protein concentration was measured using a BCA protein quantification kit (Solarbio, Beijing, China). Equal amounts of protein (50 μg) were separated by SDS–PAGE and transferred onto PVDF membranes (Whatman, Clifton, NJ, USA). After blocking with 5% milk for 2 h, the membranes were incubated with primary antibodies overnight at 4 °C. The next day, the membranes were washed three times and incubated with horseradish peroxidase-conjugated secondary antibodies (CWBIO, Beijing, China) for 1 h at room temperature. Next, the membranes were washed three times, and chemiluminescence reagent was added. The bands were visualized using ImageJ software to calculate the relative expression levels of the target protein.

### 4.5. Cell Culture and Gene Transfection

Cells were cultured in RPMI-1640 or high-glucose DMEM containing 10% fetal bovine serum (BI, Bi’ina, Israel) and incubated at 37 °C in a humidified atmosphere of 5% CO_2_. SKAP1-siRNA and NC-siRNA were purchased from Shanghai GenePharma Co., Ltd. (Shanghai, China) The GC cell lines MKN45 and HGC27 were seeded into 6-well plates at a density of 3 × 10^5^ cells/well. When cell confluence reached 60–70% the next day, TurboFect Transfection Reagent (Thermo Scientific, Waltham, MA, USA) was used to transfect SKAP1-siRNA (100 nM) or NC-siRNA (100 nM) into the cells. The sequences of siRNAs against SKAP1 are listed in Table S2. After 48 h, RNA was extracted from the cells to detect the relative expression level of SKAP1 mRNA. Protein was extracted from the cells after 72 h to detect the relative expression level of SKAP1 protein.

### 4.6. MTS Cell Proliferation Assay

After transfection for 24–48 h, cells were digested from the 6-well plates, centrifuged and resuspended to make a single cell suspension. Then, cells were seeded into 96-well plates at a concentration of 5000 cells per well in triplicate and incubated at 37 °C in a humidified atmosphere of 5% CO_2_. Every day, one 96-well plate was removed, and 20 μL of MTS reagent (Promega) was added to each well and incubated for 1.5 h. The absorbance of each well was measured at 490 nm using an enzyme-labeled instrument. After 3 days, data were processed, and cell proliferation curves were plotted for each group.

### 4.7. EdU Cell Proliferation Assay

After transfection for 24–48 h, cells were digested from six-well plates and resuspended to make a single-cell suspension. Then, the cells were seeded into 96-well plates at a density of 1.5 × 10^4^ cells per well. The next day, the medium was replaced with 50 µM EdU medium and incubated for 2 h. Cells were then fixed with 4% paraformaldehyde solution and stained with Apollo staining solution and Hoechst 33,342 staining solution. After staining, the cells were photographed using an inverted fluorescence microscope, and ImageJ software was used to analyze the cells and calculate the cell proliferation rate.

### 4.8. Colony Formation Assay

After transfection for 24–48 h, cells were digested from the 6-well plates and resuspended to make a single cell suspension. Then, cells were seeded into 6-well plates at a density of 800 cells per well. The plate was shaken in a cross shape to evenly distribute the cells and incubated at 37 °C in a humidified atmosphere of 5% CO_2_. The medium was changed when necessary. When the number of cells in each colony was approximately 50, the cells were fixed with 4% paraformaldehyde solution for 30 min and stained with 0.1% crystal violet staining solution for 30 min. After staining, the cells were photographed, and the number of cell colonies was calculated using ImageJ software.

### 4.9. Transwell Cell Invasion and Migration Assay

We performed cell invasion and migration experiments using Transwell chambers with or without matrix gel (BD Biosciences, San Jose, CA, USA). After transfection, the cells were seeded in the upper chamber of the Transwell, and the lower chamber was filled with medium containing 20% FBS. After incubation for 48 h in the chamber, the cells on the upper and lower chambers were fixed with 4% paraformaldehyde solution, stained with 0.1% crystal violet staining solution, and photographed under a microscope. The number of migrated or invaded cells was calculated using ImageJ software.

### 4.10. Flow Cytometry Apoptosis Assay

All transfected cells were collected and made into a single-cell suspension with a concentration of 1 × 10^6^ cells/mL using 1× binding buffer. Then, 100 µL of the cell suspension was mixed with 5 µL of PE and 5 µL of 7AAD staining solution for 15 min. Next, 400 µL of 1× binding buffer was added to each tube, and the data were analyzed by flow cytometry using FlowJo V10.

### 4.11. Relationship between SKAP1 Expression and Immune Infiltration in GC

The immune infiltration algorithm based on ssGSEA was applied using the R package GSVA, and the immune cell markers were obtained from a study by Bindea et al. [42]. Correlation analysis between SKAP1 expression and immune cell infiltration as well as immune checkpoint molecule expression was performed using TIMER 2.0. The TIDE algorithm for predicting potential immune therapy response was also completed using R language, and the plots were generated using the R packages ggplot2 and ggpubr.

### 4.12. Statistical Analysis

All experiments were repeated independently at least three times, and the quantitative data are expressed as the mean ± standard deviation. Independent sample (or paired) t tests were used for comparisons between two groups, and one-way ANOVA was used for multiple group comparisons if the data followed a normal distribution and had homogeneous variance. Nonparametric tests were used if the data did not meet these assumptions. GraphPad Prism 8.0 and Adobe Photoshop 2020 software were used for graphing, and SPSS 22.0 software was used for statistical analysis. *p* < 0.05 was considered to indicate statistical significance.

## Data Availability

All of the data supporting this work will be made available from the corresponding author upon reasonable request.

## Supplementary Materials

The following supporting information can be downloaded at: https://www.mdpi.com/article/10.3390/ijms241411870/s1.
