# Supplementary material for: SKAP1 Is a Novel Biomarker and Therapeutic Target for Gastric Cancer: Evidence from Expression, Functional, and Bioinformatic Analyses
Source: Int J Mol Sci. 2023 Jul 24;24(14):11870. doi: 10.3390/ijms241411870 (PMC10380396; doi:10.3390/ijms241411870)
Supplement: Supplementary file 1 [file ijms-24-11870-s001.zip › ijms-2425285-supplementary.pdf]

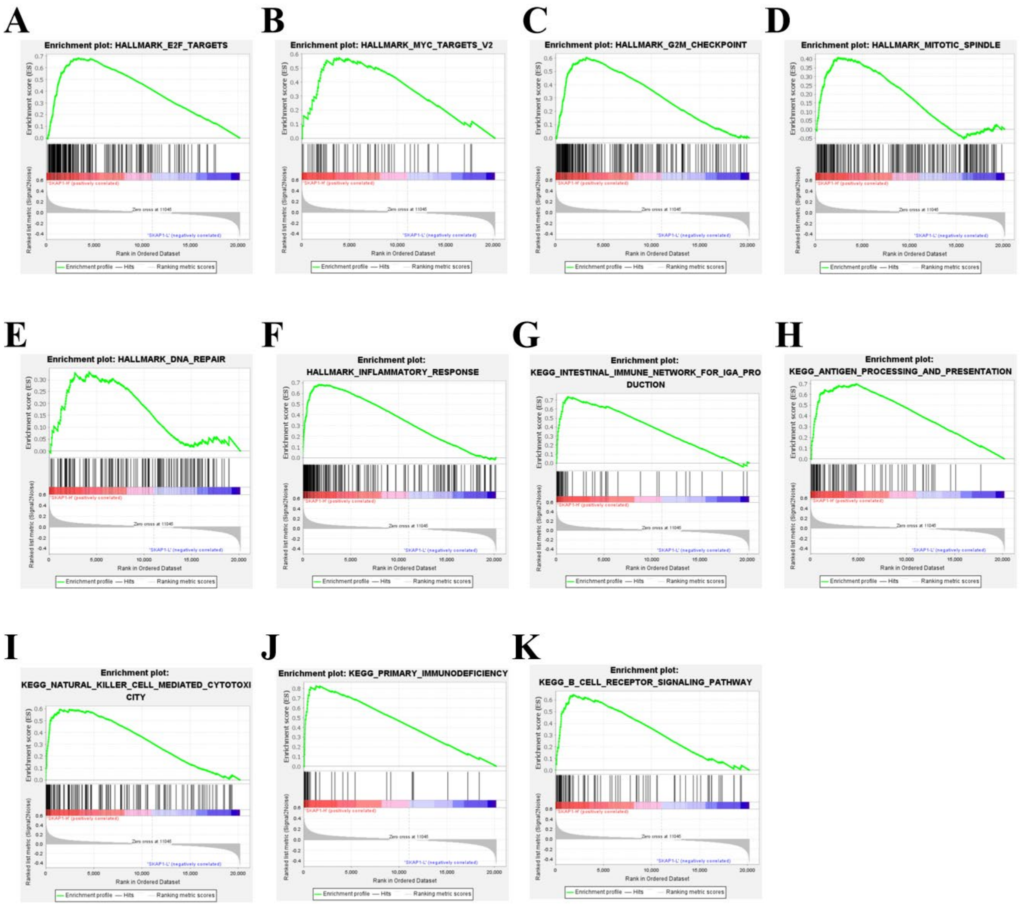

Figure S1. Gene Set Enrichment Analysis (GSEA) depicting the biological processes associated with datasets exhibiting high and low expression of SKAP1. A, E2F Targets; B, MYC Targets; C, G2M cell cycle checkpoint; D, Mtotic spindle formation; E, DNA repair; F, Inflammatory response; G, Intestinal immune network for IgA production; H, Antigen processing and presentation; I, Natural killer cell mediated cytotoxicity; J, Primary immunodeficiency; K, B cell receptor signaling pathway.

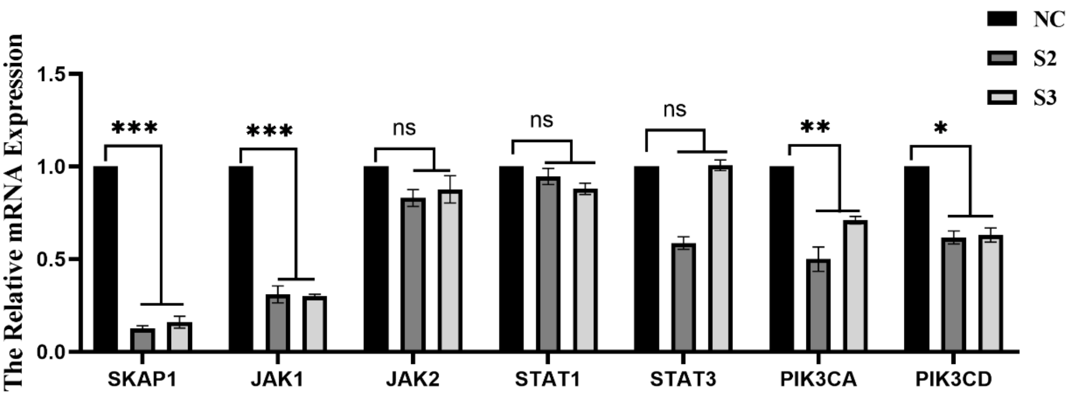

Figure S2. Quantitative Real-Time Polymerase Chain Reaction (qRT-PCR) analysis of mRNA levels of key genes within the JAK/STAT pathway and PI3K/AKT pathway following SKAP1 silencing. Negative control (NC) represents a scramble siRNA, while S2 and S3 represent siSKAP1-2, and siSKAP1-3 transfected cells, respectively. Statistical significance: The statistical significance is indicated as ns (no significance), *$P < 0.05$, **$P < 0.01$, ***$P < 0.001$. The experiment was repeated independently three times.

Table S1. Primer sequences.

Gene		Primer sequence（5′-3′）
GAPDH	F	TGACTTCAACAGCGACACCCA
GAPDH	R	CACCCTGTTGCTGTAGCCAAA
SKAP1	F	CTGCGAAGAGATTCCAAGAAAG
SKAP1	R	TGCTGGACTAGTAGCTGTAAAC
JAK1	F	CTCACCAGGATGCGGATAAA
JAK1	R	AGTTTCCAAGGTAGCCAAGTAT
JAK2	F	TCTGGGGAGTATGTTGCAGAA
JAK2	R	AGACATGGTTGGGTGGATACC
STAT1	F	GACCCAATCCAGATGTCTATGA
STAT1	R	CCCGACTGAGCCTGATTA
STAT3	F	CCTTCCTGCTAAGATTCA
STAT3	R	GACTGGATCTGGGTCTTA
PIK3CA	F	CCTGATCTTCCTCGTGCTGCTC
PIK3CA	R	ATGCCAATGGACAGTGTTCCTCTT
PIK3CD	F	CATATGTGCTGGGCATTGGC
PIK3CD	R	TTTCACAGTAGCCCCGGAAC

Table S2. siRNA sequences targeting SKAP1.

Gene		Sequence（5' -3'）
Negative control	sense	UUCUCCGAACGUGUCACGUTT
	antisense	ACGUGACACGUUCGGAGAATT
SKAP1-siRNA-1	sense	CUGAAGAGUUUCUGGCAGATT
	antisense	UCUGCCAGAAACUCUUCAGTT
SKAP1-siRNA-2	sense	GAAUGGAAGACAUCGUAAATT
	antisense	UUUACGAUGUCUUCCAUUCTT
SKAP1-siRNA-3	sense	GUUGUCAGCAGAGGUCUCUTT
	antisense	AGAGACCUCUGCUGACAACTT

Table S3. Correlation between SKAP1 and immune cell markers.

Description	Gene Markers	STAD			
		None		Purity	
		Core	*P*	Core	*P*
CD8^{+}T cell	CD8A	0.349	***	0.366	***
	CD8B	0.366	***	0.371	***
T cell (general)	CD3D	0.355	***	0.385	***
	CD3E	0.338	***	0.371	***
	CD2	0.35	***	0.383	***
B cell	CD19	0.236	***	0.255	***
	CD79A	0.232	***	0.266	***
Monocyte	CD86	0.248	***	0.277	***
	CD115 (CSF1R)	0.185	***	0.222	***
TAM	CCL2	0.1	4.11e-02	0.111	*
	CD68	0.153	***	0.184	***
	IL10	0.248	***	0.259	***
M1 Macrophage	iNOS (ISYNA1)	0.118	**	0.116	*
	iNOS (NOS2)	0.006	9.01e-01	-0.008	8.7e-01
	IRF5	0.322	***	0.352	***
	COX2 (PTGS2)	0.041	4.03e-01	0.048	3.51e-01
M2 Macrophage	CD163	0.175	***	0.198	***
	VSIG4	0.161	***	0.175	***
	MS4A4A	0.197	***	0.214	***
Neutrophils	CD66b (CEACAM8)	0.108	*	0.087	8.98e-02
	CD11b (ITGAM)	0.288	***	0.315	***
	CCR7	0.246	***	0.275	***
Natural killer cell	KIR2DL1	0.119	*	0.084	1.04e-01
	KIR2DL3	0.101	*	0.092	7.48e-02
	KIR2DL4	0.228	***	0.205	***
	KIR3DL1	0.169	***	0.157	**
	KIR3DL2	0.237	***	0.226	***
	KIR3DL3	0.051	3.04e-01	0.028	5.92e-01
	KIR2DS4	0.137	**	0.117	*
Dendritic cell	HLA-DPB1	0.321	***	0.351	***
	HLA-DQB1	0.207	***	0.223	***
	HLA-DRA	0.301	***	0.323	***
	HLA-DPA1	0.308	***	0.335	***
	BCDA-1 (CD1C)	0.178	***	0.216	***
	BCDA-4 (NRP1)	0.095	5.43e-02	0.123	*
	CD11c (ITGAX)	0.224	***	0.243	***
Th1	T-bet (TBX21)	0.332	***	0.338	***

	STAT4	0.381	***	0.411	***
	STAT1	0.274	***	0.303	***
	IFN-γ (IFNG)	0.306	***	0.306	***
	TNF-α (TNF)	0.169	***	0.19	***
Th2	GATA3	0.3	***	0.299	***
	STAT6	0.124	*	0.158	***
	STAT5A	0.372	***	0.391	***
	IL13	0.052	2.92e-01	0.057	2.7e-01
Tfh	BCL6	0.101	*	0.118	*
	IL21	0.276	***	0.279	***
Th17	STAT3	0.151	**	0.175	***
	IL17A	0.007	8.89e-01	0.024	6.42e-01
Treg	FOXP3	0.328	***	0.347	***
	CCR8	0.312	***	0.324	***
	STAT5B	0.21	***	0.25	***
	TGF-β (TGFB1）	0.202	***	0.23	***
Tex	PD-1 (PDCD1)	0.372	***	0.383	***
	CTLA4	0.342	***	0.345	***
	LAG3	0.326	***	0.339	***
	TIM-3 (HAVCR2)	0.257	***	0.284	***
	GZMB	0.256	***	0.262	***

Note: Core: Spearman’s rho value. Statistical significance: *P < 0.05, **P < 0.01, ***P < 0.001.
